# Seasonal Variation in Metabolic Syndrome and Its Association With Meteorological Factors: A Large Population-Based Study in Fukushima, 2012–2022

**DOI:** 10.1007/s44197-026-00613-z

**Published:** 2026-07-09

**Authors:** Enbo Ma, Tetsuya Ohira, Maiko Fukasawa, Atsushi Shirafuji, Kenta Matsuzaki, Yu Sato, Tohru Ohtake, Michio Shimabukuro

**Affiliations:** 1 Health Promotion Center, Fukushima Medical University, Fukushima City, 960-1295 Japan; 2 Department of Epidemiology, Fukushima Medical University School of Medicine, Fukushima City, 960-1295 Japan; 3 Radiation Medical Science Center for the Fukushima Health Management Survey, Fukushima Medical University, Fukushima City, 960-1295 Japan; 4 Department of Computer Science and Engineering, The University of Aizu, Aizuwakamatsu City, 965-8580 Japan; 5 Department of Cardiovascular Medicine, Fukushima Medical University School of Medicine, Fukushima City, 960-1295 Japan; 6 Department of Breast Surgery, Fukushima Medical University School of Medicine, Fukushima City, 960-1295 Japan; 7 Department of Diabetes, Endocrinology and Metabolism, Fukushima Medical University School of Medicine, Fukushima City, 960-1295 Japan

**Keywords:** Metabolic syndrome, Meteorological factors, Seasonal variation, Non-linear association, Generalised additive model

## Abstract

**Background:**

Seasonal variation in metabolic risk may be associated with meteorological factors, with potential implications for the prevention and control of metabolic syndrome (MetS). However, evidence on these patterns in large populations remains limited.

**Methods:**

This study included 2,913,636 health checkup records from insured adults aged 35–74 years in Fukushima, Japan, between April 2012 and March 2022. Descriptive time-series analyses were conducted to examine seasonal variation in MetS prevalence and metabolic components. Generalised estimating equation models were used to assess associations of season with MetS status and metabolic components, while generalised additive models were applied to evaluate non-linear associations between meteorological factors and MetS status.

**Results:**

Overall, 26.3% of men and 8.9% of women had MetS. Compared with spring, after adjustment for meteorological factors, the odds of having MetS were 3% and 7% higher in autumn and winter in men, and 7% and 7% higher in women, respectively. Lower temperatures were associated with higher odds of having MetS in both sexes. Sunshine duration showed sex-specific associations: both shorter and longer sunshine duration were associated with lower odds of having MetS in men, whereas longer sunshine duration was associated with higher odds of having MetS in women. Waist circumference was significantly associated with temperature in women but not in men, and showed an approximately linear association with sunshine duration in men and a J-shaped association in women. Non-linear associations between meteorological factors and other metabolic components were also observed in both sexes. In particular, temperature was inversely associated with systolic and diastolic blood pressure and high-density lipoprotein cholesterol, moderately associated with fasting plasma glucose, and showed an approximately U-shaped association with triglycerides.

**Conclusions:**

MetS status and metabolic components showed seasonal variation and were associated with meteorological conditions, particularly ambient temperature. These findings may help inform seasonal strategies for metabolic risk prevention and control.

**Supplementary Information:**

The online version contains supplementary material available at https://doi.org/10.1007/s44197-026-00613-z.

## Background

Seasonal variation, influenced by meteorological factors, has been reported in cardiovascular diseases [1–5] and type 2 diabetes mellitus [6–11] worldwide. Metabolic syndrome (MetS) is a major risk factor for these chronic diseases; however, its seasonal variation has not been extensively investigated, including in Japanese populations [1–4]. Understanding how meteorological conditions shape seasonal variation in MetS may help identify time-specific opportunities for intervention and inform strategies to reduce its burden [4, 5]. Such insights may contribute not only to the prevention and control of MetS but also to that of related chronic diseases.

Health insurance is universal in Japan. Approximately 56.3% of Fukushima residents participated in annual health checkups in 2021, similar to the national average [6]. The Fukushima Health Insurance Database (FDB) was established using individual-level health checkup data from registries in Fukushima Prefecture since 2012 [7]. The FDB includes residents from all 59 municipalities in the prefecture, and the national health insurance and employee health insurance covered 84.4% of residents aged 40–74 years in 2021 [8].

The Fukushima Health Management Survey has reported increased cardiometabolic risks among post-disaster residents, including overweight or obesity [9–11], hypertension [12–14], diabetes mellitus [9, 11], and dyslipidaemia [9, 11, 15]. Based on the FDB, previous studies have also demonstrated an increased risk of MetS over time [8, 16].

Using a large regional health checkup database in Fukushima Prefecture, we aimed to examine seasonal variation in measured MetS status and metabolic components and to evaluate whether meteorological factors were associated with this variation. In this study, season was conceptualised as a broad contextual indicator reflecting meteorological, behavioural, and physiological variation, whereas meteorological variables were examined as specific short-term environmental conditions around the time of health checkup. The analysis was intended to describe seasonal and meteorological associations with measured MetS status and metabolic components, rather than to estimate causal effects on the development of MetS. These findings may provide insights into seasonal variation in metabolic risk and inform regional health promotion strategies.

## Methods

This study used a repeated cross-sectional design based on annual health checkup records. The unit of analysis was the person-visit, defined as one health checkup record per participant per year. MetS prevalence was summarised descriptively, and MetS status was evaluated at each examination rather than as incident MetS; therefore, participants with MetS at a given examination were not excluded from subsequent analysis. Within-person correlation across repeated observations was accounted for in the GEE models.

### Study Population

The FDB participants mainly include individuals covered by national health insurance (covers unemployed, self-employed, and those in the agriculture, forestry, or fishery industry aged ≤ 74 years), employee health insurance (mainly covers employees in small and medium-sized companies and dependents aged 35–74 years), and mutual aid association health insurance (mainly covers those in administrations aged ≤ 74). This study included 2,960,868 health checkup records from FDB participants aged 35–74 years at the time of health checkup who had attended at least one health checkup between April 2012 and March 2022.

### Subareas

Owing to radiation leakage from the damaged nuclear power plant following the Great East Japan Earthquake on 11 March 2011, residents were required to relocate according to government orders. In this study, disparities in metabolic factors across merged secondary medical areas (Mountainous, Central, and Coastal areas) and evacuation areas were evaluated. The evacuation areas were classified as municipalities, which included Naraha Town, Tomioka Town, Okuma Town, Futaba Town, Namie Town, Katsurao Village, Iitate Village, Tamura City, Minami-Soma City, Kawamata Town, Hirono Town, and Kawauchi Village [17].

### Measurement of Metabolic Risk

MetS was defined according to the 2005 Japanese Diabetes Association guidelines as follows: waist circumference (WC) ≥ 85 cm in men and ≥ 90 cm in women, as well as two or more of the following three parameters: 1) triglyceride (TG) ≥ 150 mg/dL, high-density lipoprotein cholesterol (HDL-C) < 40 mg/dL, or use of anti-dyslipidaemia medication; 2) systolic blood pressure (SBP) ≥ 130 mmHg, diastolic blood pressure (DBP) ≥ 85 mmHg, or use of antihypertensive medication; and 3) elevated fasting plasma glucose (FPG) ≥ 110 mg/dL or use of diabetes therapy [18].

### Meteorological Data

Daily meteorological data were obtained from the official National Meteorological Database from April 2012 to March 2022 [19]. To avoid collinearity among available meteorological factors, in which the highest correlation coefficient was between wind and humidity (r = −0.829), the only correlation > 0.80, we excluded wind and included daily means of ambient temperature (^0^C), relative humidity (%), precipitation (mm), atmospheric pressure (station level) (hPa), and sunshine duration (hours). Precipitation was defined as total daily rainfall (mm) and did not include snowfall. A preliminary analysis including snowfall showed negligible differences in estimates; therefore, only rainfall was used in the final analysis.

Meteorological variables were assigned according to the date of each participant’s health checkup. Same-day exposure was defined as the meteorological value on the checkup date. Moving-average meteorological values were calculated for the checkup date and the preceding 3 days (MA0–3) or 7 days (MA0–7), respectively, and were used in the GAM sensitivity analyses.

### Statistical Analysis

After removing 47,222 records with missing or extreme values of metabolic factors (WC < 50 cm or WC > 200 cm, SBP < 70 mmHg or SBP > 270 mmHg, DBP < 40 mmHg or DBP > 150 mmHg, FPG < 40 mg/dL or FPG > 500 mg/dL, HDL-C < 5 mg/dL or HDL-C > 150 mg/dL, and TG < 10 mg/dL or TG > 3000 mg/dL), 2,913,636 health checkup records/person-visits from individuals with at least one health checkup were included in the analysis. The excluded records accounted for approximately 1.6% of the initial records. Annual MetS prevalence was computed based on the individual metabolic component factors at each health checkup, and trends in MetS prevalence were assessed across calendar years.

First, we decomposed time-series data on metabolic risk into secular trends, seasonal variations, and residual components using an additive adjustment in an unobserved components model. The additive form of the model was specified as follows:

*Yt=TCt+St+It,*

where t = 1, 2, …, n denotes time, *Yt* denotes the observed series, *TCt* is the trend-cycle component (level + trend), *St* denotes the seasonal component, and *It* is the irregular variation (error term). To visualise seasonal patterns, the identified trend component *(TCt)* with the mean of observations or the integrated monthly average means of MetS and the component.

factors, along with their 95% confidence intervals, were plotted over time.

Population-averaged associations between seasons and MetS status and metabolic component factors were estimated using generalised estimating equations (GEE), accounting for repeated measurements within individuals [20]. The GEE analyses were restricted to individuals with at least two health checkups during the study period, resulting in 2,693,687 records/person-visits after excluding 219,949 records from individuals with only one health checkup. GEE models were used to account for within-person correlation among repeated health checkup records. The four seasons were classified as spring (March–May), summer (June–August), autumn (September–November), and winter (December–February). Model 1 was adjusted for age group (35–39, 40–44, 45–49, 50–54, 55–59, 60–64, 65–69, and 70–74 years), current smoking (no, yes), alcohol drinking (almost no, sometimes, and every day), evacuation (no, yes), residential areas (mountainous, central, coastal, and other prefectures), weekly moderate-intensity exercise (defined as ≥ 2 times/week, ≥ 30 min/session; no, yes), daily physical activity or walking (defined as ≥ 1 h/day; no, yes), hypertension, hyperlipidaemia, or diabetes therapy (no, yes), and history of cardiovascular or kidney disease (no, yes).

The five meteorological variables were selected a priori based on epidemiological relevance and data availability. Because season and meteorological factors are closely correlated, models including both were interpreted cautiously and were not intended to fully separate independent effects. In the GEE analysis, Model 1 included season and covariates, and Model 2 was additionally adjusted for same-day temperature (^0^C), precipitation (mm), sunshine duration (hours), relative humidity (%), and atmospheric pressure (hPa). Variance inflation factors (VIFs) were assessed in an auxiliary model including season and the five same-day meteorological variables; no severe multicollinearity was observed, with all VIFs below 4.30. Because MA0–3 and MA0–7 variables showed higher collinearity with season, they were not included in the main GEE models.

In the GAM analysis, meteorological variables were the exposures of interest, and season was included to adjust for broad residual seasonal patterns. To assess non-linear exposure–response patterns, generalised additive models (GAMs) were fitted with penalised regression splines for each meteorological factor. For each factor, the maximum degrees of freedom (*maxdf*) for spline terms were selected based on the lowest Bayesian Information Criterion (BIC) from all possible 3–5 *df* combinations, separately for men and women. When BIC values differed by ≤ 2, the simpler model was chosen. An effective degree of freedom (EDF) of approximately 1 indicates an approximately linear effect on the logit scale; larger EDF values reflect increasing non-linearity. In the GAM analysis, same-day meteorological variables were used as the primary exposure window. MA0–3 and MA0–7 exposures were examined as sensitivity analyses to assess whether non-linear associations between meteorological conditions and measured MetS status were robust to short-term cumulative exposure windows. These results were presented in the supplementary material. Season-stratified GAM analyses using same-day meteorological variables were also conducted to examine whether the meteorological associations were observed within seasons.

Analyses were stratified by sex. Statistical analyses were performed using SAS software, version 9.4 (SAS Institute Inc., Cary, NC). Two-sided *P* values were reported, with values < 0.05 considered statistically significant.

## Results

Table 1 presents the characteristics of health checkup participants by season from April 2012 to March 2022. Differences were observed across seasons for all variables (*P* values < 0.001). Most health checkups were conducted in summer and autumn, with higher proportions among men (69.6%) and women (72.5%). For both sexes, the rates of evacuation, exercise, and physical activity, as well as history of cardiovascular and kidney diseases, were higher in summer and autumn than in spring and winter. Smoking prevalence was lower in summer and autumn than in spring and winter. Meanwhile, the prevalence of daily alcohol consumption was higher in men but lower in women in summer and autumn than in spring and winter. Men had a much higher prevalence of MetS (26.3%) than women (8.9%), both overall and across seasons. Women had more favourable metabolic indicators than men, with lower WC, TG, FPG, SBP, and DBP levels and higher HDL-C levels, with fewer individuals receiving hypertension and diabetes therapies, except for those receiving hyperlipidaemia therapy.

**Table 1:** Characteristics of participants with health checkups and meteorological measurements in each season, 2012–2022

X	All	Spring (Mar-May)	Summer (Jun-Aug)	Autumn (Sep-Nov)	Winter (Dec-Feb)
Men, *n* (%)	1 467 802 (100)	237 631 (16.2)	522 782 (35.6)	498 423 (34.0)	208 966 (14.2)
Age, year	56.6 ± 11.5	53.5 ± 10.8	58.1 ± 11.5	57.6 ± 11.5	54.2 ± 11.1
Current smoking, %	38.0	43.3	36.5	35.8	40.9
Alcohol drinking					
Every day, %	40.4	39.4	41.4	40.5	38.9
Sometimes, %	25.3	26.1	24.6	25.2	26.6
Evacuation, %	12.0	9.4	12.6	13.4	10.5
Moderate-density exercise ≥ 2 times/week, %	26.7	20.6	29.6	28.3	22.3
Physical activity or walking ≥ 1 h/day, %	35.8	32.3	37.1	36.4	35.2
Resident area					
Mountain, %	14.8	14.0	16.9	15.3	9.4
Central, %	56.8	54.5	58.6	56.1	56.9
Coastal, %	22.7	24.8	19.8	23.1	26.5
WC, cm	85.6 ± 9.5	85.7 ± 9.4	85.5 ± 9.7	85.4 ± 9.4	85.7 ± 9.7
BMI (kg/m^2^)	24.2 ± 3.6	24.3 ± 3.7	24.1 ± 3.5	24.2 ± 3.5	24.4 ± 3.7
SBP, mmHg	128.3 ± 16.1	129.0 ± 16.1	128.1 ± 16.2	127.3 ± 15.9	129.4 ± 16.4
DBP, mmHg	78.6 ± 11.3	78.8 ± 11.2	79.3 ± 11.5	77.6 ± 11.2	79.9 ± 11.5
TG, mg/dL	105 (74, 154)	103 (72, 154)	105 (74, 154)	105 (74, 154)	105 (73, 156)
HDL-C, mg/dL	55 (46, 65)	56 (47, 66)	54 (46, 64)	55 (47, 65)	56 (47, 67)
FPG, mg/dL	98 (91, 108)	98 (91, 107)	98 (91, 107)	98 (91, 108)	99 (92, 109)
History of cardiovascular or kidney disease, %	7.6	6.2	8.2	8.2	6.7
Hypertension therapy, %	30.9	25.7	33.0	32.4	27.6
Hyperlipidemia therapy, %	15.0	12.5	15.7	15.8	13.9
Diabetes therapy, %	8.9	7.3	9.4	9.2	8.4
MetS, %	26.3	24.4	26.2	27.0	26.9
Women, *n* (%)	1 445 834 (100)	168 854 (11.7)	507 447 (35.1)	540 461 (37.4)	229 072 (15.8)
Age, years	58.2 ± 11.0	59.4 ± 10.7	54.1 ± 10.8	60.0 ± 10.7	54.1 ± 10.7
Current smoking, %	10.9	14.3	9.5	10.1	12.2
Alcohol drinking					
Every day, %	10.4	11.8	9.5	10.1	12.2
Sometimes, %	24.1	25.8	23.0	23.3	27.2
Evacuation, %	12.6	9.9	13.7	13.0	10.8
Resident area					
Mountain, %	16.4	17.9	19.3	16.9	7.8
Central, %	58.0	55.3	59.2	56.9	60.3
Coastal, %	21.8	21.7	18.6	22.6	26.8
Moderate-density exercise ≥ 2 times/week, %	22.3	14.6	25.7	24.4	15.6
Physical activity or walking ≥ 1 h/day, %	32.1	27.7	32.9	32.7	31.8
WC, cm	81.0 ± 10.3	81.4 ± 10.2	80.2 ± 10.5	81.1 ± 10.2	80.5 ± 10.5
BMI (kg/m^2^)	23.0 ± 3.9	23.0 ± 4.0	22.9 ± 3.8	23.0 ± 3.9	23.0 ± 4.0
SBP, mmHg	124.2 ± 16.7	125.4 ± 16.7	122.9 ± 16.9	123.6 ± 16.5	123.7 ± 16.7
DBP, mmHg	74.0 ± 10.9	74.5 ± 10.8	74.0 ± 11.3	73.2 ± 10.8	74.4 ± 11.2
TG, mg/dL	82 (60, 114)	75 (55, 106)	84 (61, 116)	84 (61, 116)	77 (56, 109)
HDL-C, mg/dL	66 (56, 77)	68 (58, 79)	64 (55, 75)	66 (56, 77)	69 (58, 80)
FPG, mg/dL	93 (88, 100)	93 (87, 100)	93 (88, 100)	93 (87, 101)	93 (87, 101)
History of cardiovascular or kidney disease, %	4.2	3.3	4.5	4.6	3.3
Hypertension therapy, %	25.1	18.8	27.6	27.1	19.3
Hyperlipidemia therapy, %	20.7	14.5	23.4	22.6	15.0
Diabetes therapy, %	4.5	3.5	4.8	4.8	3.7
MetS, %	8.9	7.0	9.1	9.7	7.8
Meteorological factor (daily, median (IQR))					
Temperature, ^0^C	18.5 (10.1, 22.8)	13.9 (7.8, 18.2)	24.0 (21.5, 26.3)	16.0 (12.3, 20.7)	3.4 (1.7, 4.3)
Relative Humidity, %	72.7 (66.8, 78.0)	60.0 (57.4, 64.2)	75.3 (71.0, 80.8)	75.0 (69.0, 79.0)	70.0 (66.2, 73.0)
Precipitation, mm	1.7 (0.0, 4.5)	2.2 (1.4, 3.0)	2.7(0.0, 6.3)	1.0 (0.0, 5.0)	1.3 (0.5, 2.0)
Sunshine duration, hour	4.8 (3.1, 6.1)	6.5 (5.7, 7.3)	4.8 (2.2, 6.1)	4.2 (2.7, 5.6)	4.3 (3.5, 5.1)
Atmospheric pressure, hPa	1005.6 (1001.7, 1009.1)	1005.2 (1002.6, 1006.8)	1001.5 (1000.0,100.92)	1008.4 (1005.7, 1011.2)	1008.2 (1006.3, 1010.2)

^*^Measurements were shown in mean (SD) or medians (IQR). BMI, body mass index; DBP, diastolic blood pressure; FPG, fasting plasma glucose; HDL-C, high-density lipoprotein cholesterol; IQR, interquartile range; MetS, metabolic syndrome; SBP, systolic blood pressure; SD, standard deviation; TG, triglyceride; WC, waist circumference

Table 1 also presents meteorological measurements overall and for each season. Figure S1 illustrates the monthly median (IQR) of daily temperature, relative humidity, precipitation, atmospheric pressure, and sunshine duration in Fukushima from April 2012 to March 2022. Meteorological factors were correlated (Table S1). The highest positive correlation was observed between precipitation and relative humidity (r = 0.447), whereas the lowest negative correlation was found between sunshine duration and relative humidity (r = −0.704).

**Fig. 1:**
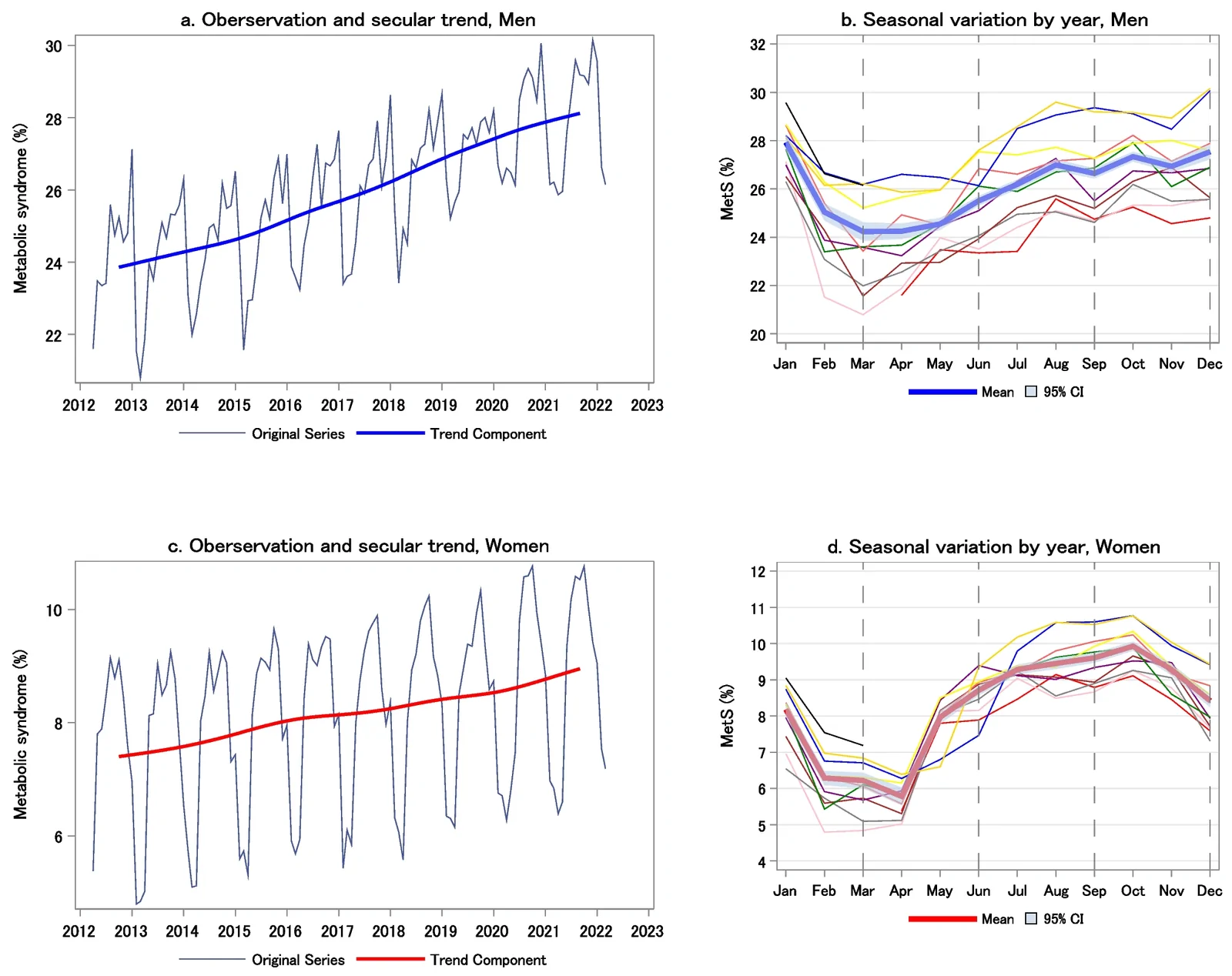
Seasonal variation and secular trends in the prevalence of metabolic syndrome in men and women with annual health checkups, April 2012– March 2022. Panels (**a**) and (**c**) show secular trends with smoothers; panels (**b**) and (**d**) show seasonal patterns based on month-specific means over years (95% confidence intervals)

No increasing trend in MetS prevalence was observed in men (*P* = 0.074) or women (*P* = 0.595), whereas seasonal variation was detected in both men and women (*P* < 0.001) (Fig. 1 (a) and (c)). In the observed data, MetS prevalence in men was high in winter; in both sexes, it was high in late summer and early autumn and low in spring (Fig. 1 (b) and (d)). Variation in MetS prevalence was also visualised in year-by-month-dimensional graphs, which aided simultaneous assessment of secular and seasonal trends (Figure S2).

**Fig. 2:**
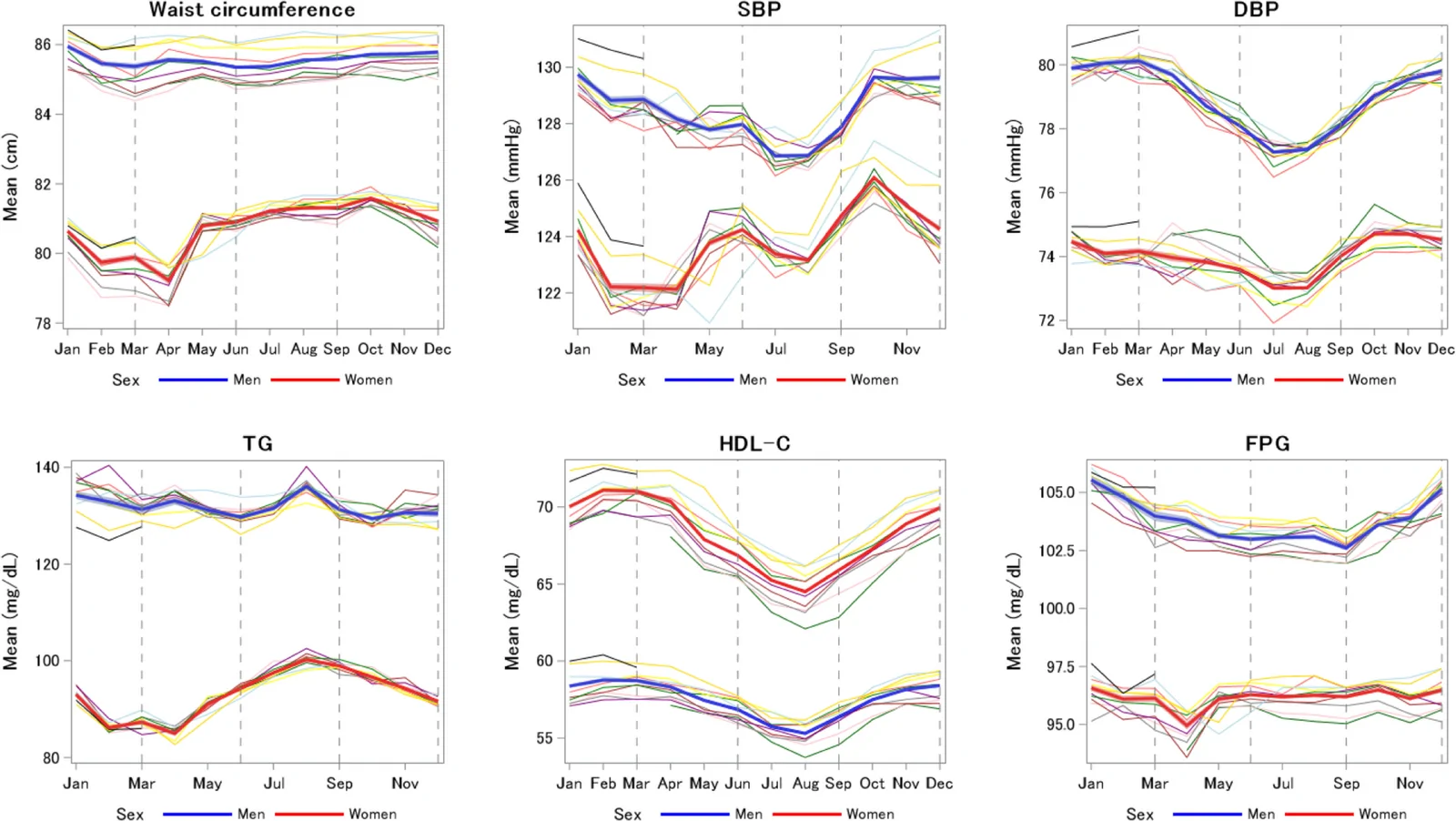
Monthly mean values (with 95% confidence intervals) of metabolic components by sex, 2012–2022. Panels show waist circumference (cm), systolic blood pressure (mmHg), diastolic blood pressure (mmHg), triglycerides (mg/dL), HDL-cholesterol (mg/dL), and fasting plasma glucose (mg/dL). Thick lines represent monthly means pooled across all study years; shaded bands indicate 95% confidence intervals of the monthly estimates. Separate y-axis scales are used for each measurement to improve visualisation of seasonal variation

Men and women exhibited different patterns of variation in metabolic component factors (Fig. 2**)**; both had the highest TG levels and the lowest DBP and HDL-C levels in August. The levels of WC, SBP, TG, and FPG in spring were lower than those in other seasons in women but not in men. Seasonal variation was also observed in the prevalence of abnormal metabolic component measurements, with a pronounced increase in the prevalence of high WC and FPG and a decline in the prevalence of low HDL-C in both men and women (Figure S3A and S3B).

Compared with spring, autumn and winter were associated with higher odds of measured MetS in both sexes in Model 1. These associations were attenuated after additional adjustment for same-day meteorological factors in Model 2, particularly for winter, but remained statistically significant. Summer showed no clear association in either model. Compared with spring, the odds of measured MetS were 3–7% higher in men and approximately 7% higher in women in autumn and winter after adjusting for same-day meteorological factors (Table 2).

**Table 2:** Associations (OR (95% CI)) between seasons and metabolic syndrome, GEE analysis, 2012–2022

	Model	Meteorological factors adjustment	Spring (Mar-May)	Summer (Jun-Aug)	Autumn (Sep-Nov)	Winter (Dec-Feb)
Men	1	-	Ref	0.99 (0.97,1.01)	1.04 (1.02,1.06)	1.13 (1.10,1.15)
	2	Day 0	Ref	1.01 (0.98,1.03)	1.03 (1.01,1.05)	1.07 (1.04,1.10)
Women	1	-	Ref	0.99 (0.96,1.03)	1.09 (1.05,1.13)	1.14 (1.10,1.19)
	2	Day 0	Ref	1.01 (0.97,1.05)	1.07 (1.04,1.11)	1.07 (1.03,1.12)

^*^OR, odds ratio; CI, confidence interval. Model 1 was adjusted for age group (35–39, 40–44, 45–49, 50–54, 55–59, 60–64, 65–69, and 70–74 years), current smoking (no, yes), alcohol drinking (almost no, sometimes, and every day), evacuation (no, yes), areas (mountain, central, coastal, and other prefectures), weekly moderate exercises (≥ 2 times/week, ≥ 1 h/session; no, yes), daily physical activity or walking (≥ 1 h/day; no, yes), hypertension, hyperlipidemia, or diabetes therapy (no, yes), and history of cardiovascular or kidney disease (no, yes). Model 2, further adjusted for daily temperature (^0^C), relative humidity (%), precipitation (mm), sunshine duration (hour), and current atmospheric pressure (hPa). Model 2 showed a substantially lower AIC (a reduction in AIC of approximately 100 compared Model 1)

For metabolic risk factors, higher SBP, DBP, and HDL-C levels were observed in winter than in spring in Model 1 (Figure S4). However, after additional adjustments for meteorological factors (Model 2), these seasonal differences were attenuated or reversed suggesting that seasons and meteorological variables captured overlapping information.

Table 3 shows the smoothing component statistics for each meteorological factor in Model 2, determined by BIC minimisation (*maxdf* in men: 3/3/3/3/3 and in women: 4/3/4/3/3 for temperature, precipitation, humidity, sunshine, and pressure, respectively). Significant smooth components were observed between temperature and sunshine duration in men, and between temperature, precipitation, relative humidity, and sunshine duration in women. Results using MA0–3 and MA0–7 meteorological exposures in the GAM analyses are shown in Supplementary Table 2. The non-linear patterns were generally consistent with those based on same-day meteorological exposures, suggesting that the GAM findings were not limited to the single-day exposure definition. However, these moving-average analyses were interpreted as sensitivity analyses of short-term cumulative meteorological conditions, not as long-term exposure analyses.

**Table 3:** Smooth components statistics for associations between meteorological factors and metabolic syndrome, GAM analysis, 2012–2022

Sex	Spline Components	EDF^#^	Chi-Square	*P*-value	No of knots	*maxdf*
Men	Temperature (^0^C)	1.99	68.86	< 0.001	461	3
	Precipitation (mm)	1.00	2.77	0.096	220	3
	Relative humidity (%)	2.00	25.55	< 0.001	181	3
	Sunshine duration (hour)	1.98	41.63	< 0.001	257	3
	Atmospheric pressure (hPa)	2.00	3.21	0.201	458	3
Women	Temperature (^0^C)	2.00	79.66	< 0.001	460	4
	Precipitation (mm)	1.00	11.02	< 0.001	220	3
	Relative humidity (%)	2.00	82.16	< 0.001	181	4
	Sunshine duration (hour)	1.01	22.22	< 0.001	257	3
	Atmospheric pressure (hPa)	1.99	12.79	0.002	457	3

^*^The model 2 results, adjusted for age group (35–39, 40–44, 45–49, 50–54, 55–59, 60–64, 65–69, and 70–74 years), current smoking (no, yes), alcohol drinking (almost no, sometimes, and every day), evacuation (no, yes), areas (mountain, central, coastal, and other prefectures), weekly moderate exercises (≥ 2 times/week, ≥ 1 h/session; no, yes), daily physical activity or walking (≥ 1 h/day; no, yes), hypertension, hyperlipidemia, or diabetes therapy (no, yes), history of cardiovascular or kidney disease (no, yes), and seasons (spring, summer, autumn, and winter)

^#^EDF, effective degrees of freedom. Maximum degrees of freedom (*maxdf*) for each spline were selected by minimising the Bayesian information criterion (BIC) across all 3–5 *df* combinations. When BIC differences were ≤ 2, the simpler model was chosen

**Fig. 3:**
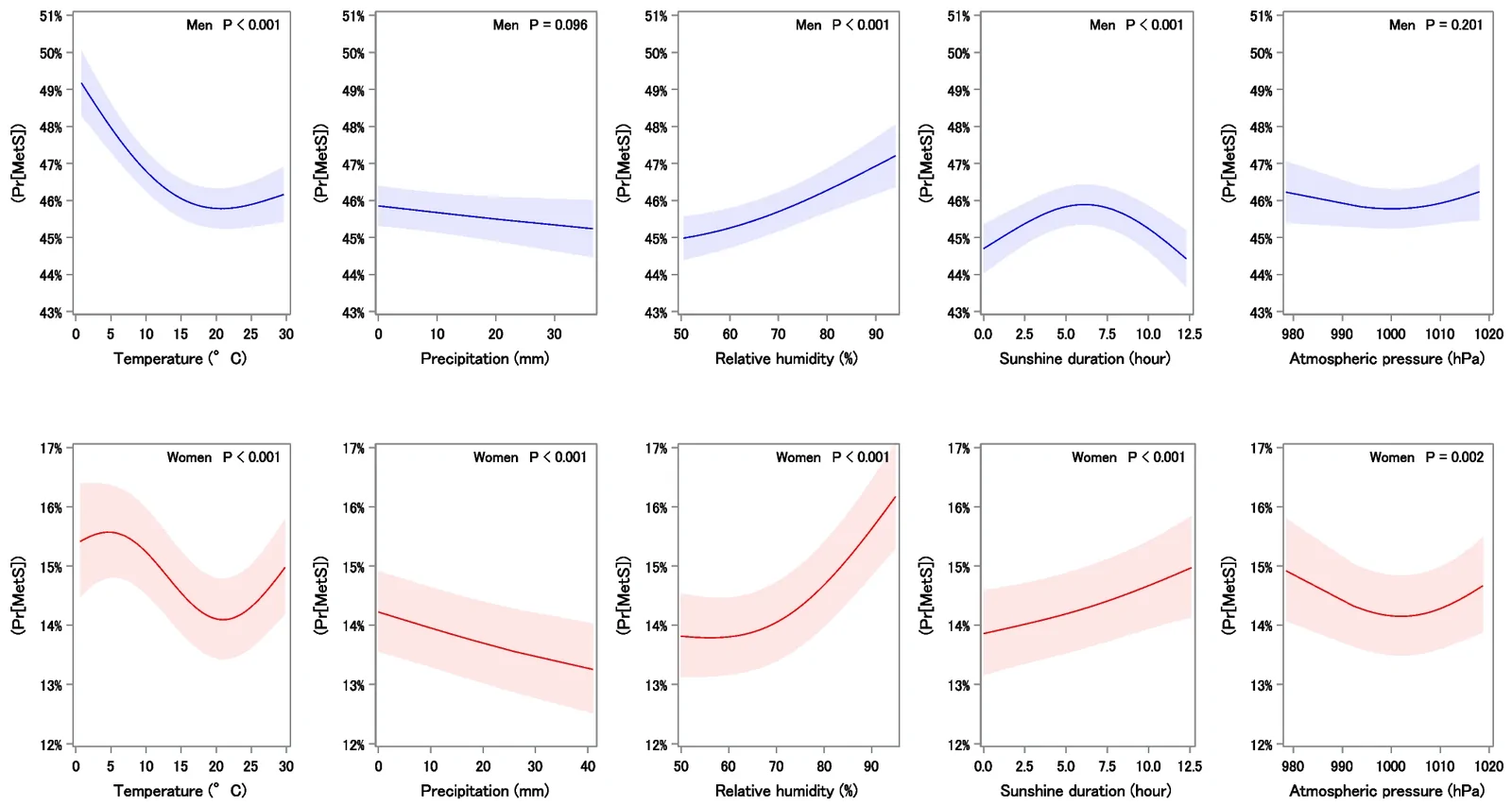
Smoothed associations between same-day meteorological factors and measured metabolic syndrome status, by sex. Curves show predicted probabilities from generalised additive models; shaded areas indicate 95% confidence intervals. Panels are shown separately for men and women. Study period: April 2012–March 2022

Figure 3 illustrates the predicted probability of measured MetS according to each same-day meteorological factor based on the GAM models, separately for men and women. Among men, temperature, relative humidity, and sunshine duration showed clearer associations with measured MetS status, consistent with the significant smooth-component tests. Precipitation showed only a borderline association and the curve was nearly flat, while atmospheric pressure showed a weak and non-significant pattern. Among women, all five meteorological factors were significantly associated with measured MetS status, with non-linear patterns observed for temperature, relative humidity, sunshine duration, and atmospheric pressure, whereas the association with precipitation was more modest.

Supplementary Figures S5–S10 show predicted spline curves from the GAM analysis for WC, SBP, DBP, log-transformed TG, HDL-C, and log-transformed FPG, respectively, separately for men and women. These component analyses support the interpretation that proximal meteorological conditions may be associated with measured MetS classification partly through short-term variation in selected metabolic components, particularly blood pressure and selected laboratory measures, rather than through acute change in waist circumference.

In season-stratified analyses, associations between meteorological factors and MetS were also observed within seasons, although the patterns and statistical significance varied by season and sex (Table S3). In men, significant non-linear associations were observed for temperature across all seasons, while associations for precipitation, humidity, sunshine duration, and atmospheric pressure were present only in selected seasons. In women, significant non-linear associations were more generally evident in summer and winter, whereas findings in spring and autumn were less consistent.

## Discussion

This study examined seasonal variation in measured MetS status and metabolic components using large regional data in Japan, as well as non-linear associations of MetS status and metabolic components with major meteorological factors.

Trends in annual MetS prevalence from 2012 to 2022 were consistent in the aggregate slope test and individual-level GEE analyses. MetS prevalence increased between 2012 and 2019 [16], but the trend slowed after 2019 in both men (*P* for trend = 0.896) and women (*P* for trend = 0.926).

Same-day meteorological exposure was selected as the primary exposure window because the metabolic components used to define MetS were measured at the time of health checkup. MA0–3 and MA0–7 exposures were examined in the GAM analyses as sensitivity windows for short-term cumulative meteorological conditions, rather than as long-term exposure metrics. Therefore, the present findings should be interpreted as descriptive associations between season, proximal meteorological conditions, and measured MetS status at health checkups, rather than as causal effects on MetS development. Proximal meteorological conditions may be associated with measured MetS classification through short-term variation in selected components, particularly blood pressure and, to a lesser extent, FPG and TG, as suggested by previous studies [21, 22]. In contrast, waist circumference is unlikely to change acutely and mainly reflects chronic adiposity. Studies using longer-term exposure metrics or seasonal averages are needed to evaluate long-term aetiological effects of meteorological conditions on MetS development.

Season and meteorological factors should be interpreted as related but distinct constructs. Season may reflect broad contextual differences, including weather, diet, physical activity, sleep, heating or cooling practices, and physiological adaptation. In contrast, same-day meteorological variables represent proximal environmental conditions around the examination date. Because several seasonal behaviours were not measured directly, the meteorological estimates should not be interpreted as fully explaining the observed seasonal differences in MetS.

The GAM analyses suggested non-linear associations between meteorological factors and MetS status. These patterns may reflect combined influences of thermoregulatory stress, seasonal changes in blood pressure, physical activity, diet, hydration, and other unmeasured behaviours. However, these curves should be interpreted cautiously because the study was not designed to identify specific meteorological thresholds. Threshold estimation would require prespecified models and validation in independent datasets. The broadly consistent findings in the MA0–3, MA0–7, and season-stratified GAM analyses support the robustness of the main patterns.

The observed associations were modest in magnitude and should be interpreted as small population-level differences in measured MetS status rather than large individual-level effects. A similar study conducted in Osaka City based on national health insurance and later elderly health insurance data, where summers are very hot and humid and winters moderately cold and dry, showed higher MetS prevalence in winter, consistent with our results, and a more prominent increase in summer than that observed in our study for both sexes [4]. The authors suggested that the long summer vacation may have increased the risk of MetS [4]. Global studies have shown that high-calorie dietary intake coupled with reduced physical activity in winter leads to increases in body weight [23, 24] and BMI [25].

Temperature also showed prominent associations with metabolic risk components. Exposure to cold temperatures may increase sympathetic nervous system activation and vasoconstriction, and reduce endothelial function, thereby contributing to increased blood pressure [26, 27]. Without meteorological adjustment, SBP and DBP were higher in winter than in spring (Model 1). After adjusting for ambient temperature (Model 2), these differences were attenuated, indicating that lower temperatures largely explained the winter increase in blood pressure and seasonal coefficients were reduced when correlated meteorological variables were included.

Conversely, higher ambient temperatures may reduce adaptive thermogenesis and physical activity, lower energy expenditure, and increase the prevalence of obesity and MetS [28, 29]. In our study, higher temperatures were positively associated with WC in women and with higher TG levels and lower HDL-C levels in both sexes. To control MetS, interventions should emphasize balanced diets that account for seasonal changes in dietary intake, meal frequency, and nutritional status [24, 30–32].

Our study indicated that higher temperatures were associated with lower FPG levels, similar to the Osaka study [4], but with less variation in summer. Our results are also consistent with those of a Chinese study in Kailuan [31], located in a similar latitude zone as Fukushima. Another large Chinese cross-sectional study [33] and a meta-analysis [34] reported that FPG levels were higher in winter than in summer and were higher in cold areas. It has been reported that a 10 ^0^C increase in mean annual temperature was associated with a 33% higher risk of developing elevated FPG [35]. However, we did not observe an increase in temperature in Fukushima during the study period (*P* > 0.062). In addition, variations in HDL-C levels showed patterns similar to those reported in the meta-analysis [34]. However, TG levels were higher both in summer and winter in our study (GAM analysis), as reported in other studies [36].

One strength of this study is the use of a multiple analytical approach. In addition, our study was based on a real-world database, enhancing the robustness of our findings. Because this study focused on prefecture-level associations, we used prefecture-level averages pooled across the available stations rather than spatial interpolation.

Several limitations should be noted. First, meteorological exposure was assigned using prefecture-level averaged data and was not linked to individual residential locations or municipality-level meteorological observations. This may have introduced exposure misclassification, particularly if meteorological conditions varied spatially within Fukushima Prefecture. Such misclassification may have attenuated or distorted the observed associations. Second, we examined short-term cumulative meteorological conditions using moving averages in the sensitivity analyses of GAM models. However, this approach did not precisely account for lag structures [37], despite the availability of daily weather information. This was because the current FDB did not have a complete list of health checkup dates for all insurance types, which limited the construction of detailed daily exposure histories. Third, the distribution of health checkups was uneven across seasons, with more conducted in summer and autumn and fewer during vacation periods, holidays, and the beginning or end of a fiscal year. This seasonal imbalance in measurement timing may have introduced structural bias and influenced the observed seasonal patterns in measured MetS status and metabolic components. Therefore, the seasonal associations should not be interpreted as reflecting biological seasonality alone. Participants who underwent health checkups may also have had higher health consciousness or different demographic profiles than those who did not. Although the models were adjusted for measured demographic, lifestyle, and clinical covariates, selection bias cannot be excluded, and the findings may have limited generalisability to other populations. Fourth, we included participants receiving medications for hypertension, hyperlipidaemia or type 2 diabetes, which may have attenuated variations in metabolic components; for instance, these medications may be more frequently prescribed in winter [4, 38]. Furthermore, information on important potential confounders, including dietary intake or behaviour, socioeconomic status, detailed physical activity, sleep, hydration, heating or cooling practices, and environmental co-exposures, was not available in the health checkup database. Therefore, residual confounding cannot be excluded, and unmeasured seasonal behavioural or environmental factors may have partly contributed to the observed associations.

## Conclusions

MetS status and metabolic components showed seasonal variation and were associated with selected meteorological factors, particularly ambient temperature. The observed associations were modest in magnitude and should be interpreted as small population-level differences in measured MetS status rather than large individual-level effects. These findings may provide insights into seasonal variation in metabolic risk and inform regional health promotion strategies.

## Supplementary Information

Below is the link to the electronic supplementary material.

Supplementary file1 (PDF 43 KB)

Supplementary file2 (PDF 66 KB)

Supplementary file3 (PDF 49 KB)

Supplementary file4 (PDF 49 KB)

Supplementary file5 (PDF 49 KB)

Supplementary file6 (PDF 58 KB)

Supplementary file7 (PDF 57 KB)

Supplementary file8 (PDF 57 KB)

Supplementary file9 (PDF 57 KB)

Supplementary file10 (PDF 58 KB)

Supplementary file11 (PDF 57 KB)

Supplementary file12 (DOCX 18 KB)

Supplementary file13 (DOCX 24 KB)

Supplementary file14 (DOCX 29 KB)

## Data Availability

The datasets generated and/or analysed during the current study are not publicly available due to confidentiality restrictions; however, they are available from the corresponding author upon reasonable request and with permission from the Fukushima Prefectural Government.
