# Supplementary material for: Seasonal Variation in Metabolic Syndrome and Its Association With Meteorological Factors: A Large Population-Based Study in Fukushima, 2012–2022
Source: J Epidemiol Glob Health. 2026 Jul 9;16(1):126. doi: 10.1007/s44197-026-00613-z (PMC13638822; doi:10.1007/s44197-026-00613-z)
Supplement: Supplementary file 3 [file 44197_2026_613_MOESM1_ESM.pdf]

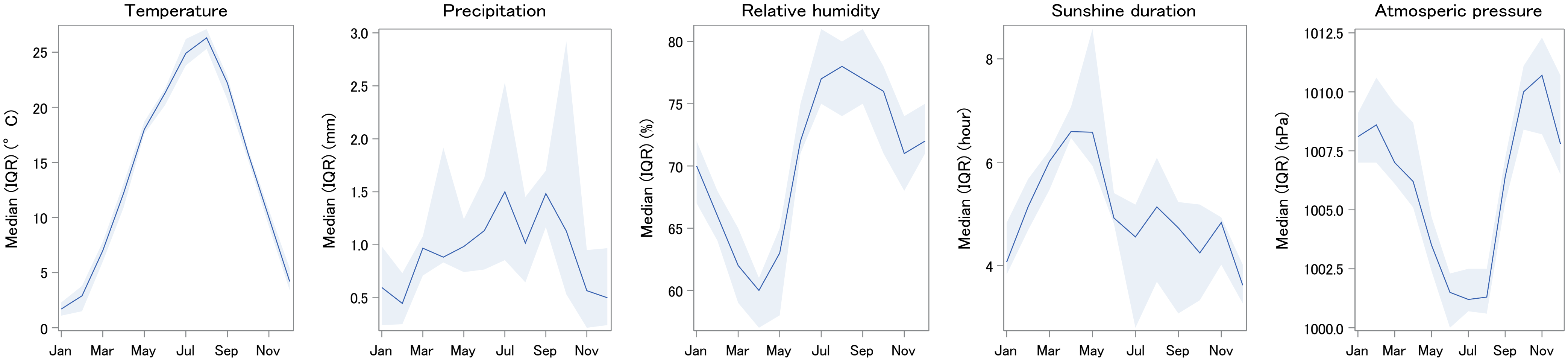

Figure S1. Monthly median (IQR) of daily temperature, relative humidity, precipitation, sunshine duration, and atmospheric pressure of Fukushima, 2012 April–2022 March.
