# Supplementary material for: Seasonal Variation in Metabolic Syndrome and Its Association With Meteorological Factors: A Large Population-Based Study in Fukushima, 2012–2022
Source: J Epidemiol Glob Health. 2026 Jul 9;16(1):126. doi: 10.1007/s44197-026-00613-z (PMC13638822; doi:10.1007/s44197-026-00613-z)
Supplement: Supplementary file 4 [file 44197_2026_613_MOESM2_ESM.pdf]

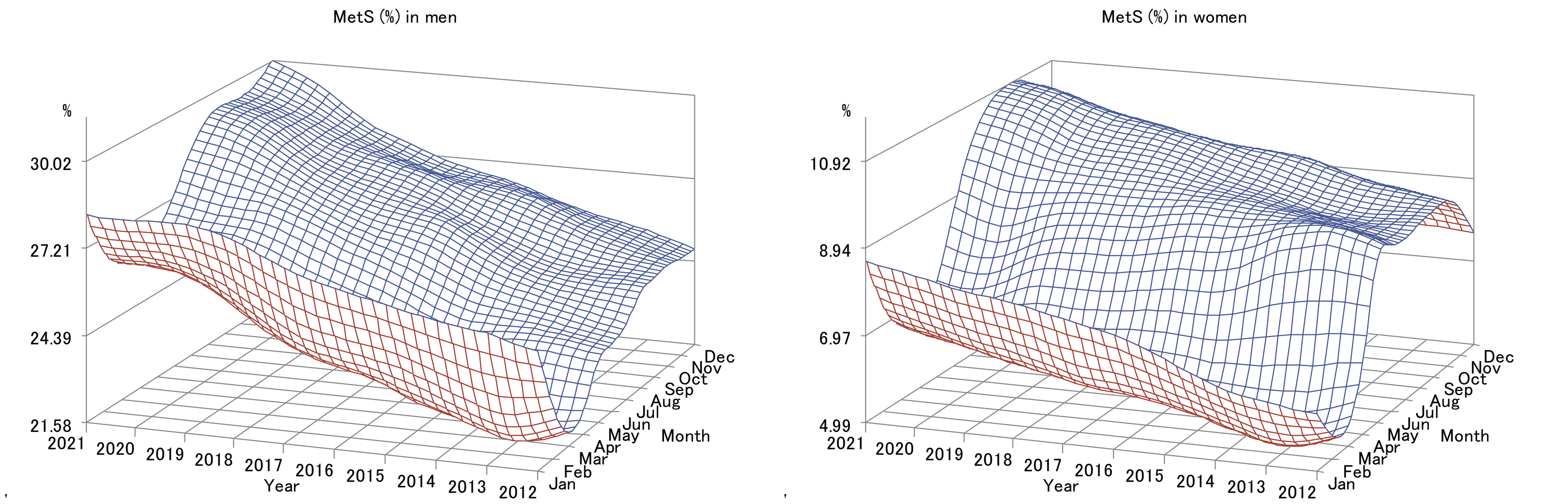

Figure S2. Three dimensions of proportions of metabolic syndrome by year and month in men (a) and women (b), 2012 April–2022 March.
