# Supplementary material for: Seasonal Variation in Metabolic Syndrome and Its Association With Meteorological Factors: A Large Population-Based Study in Fukushima, 2012–2022
Source: J Epidemiol Glob Health. 2026 Jul 9;16(1):126. doi: 10.1007/s44197-026-00613-z (PMC13638822; doi:10.1007/s44197-026-00613-z)
Supplement: Supplementary file 7 [file 44197_2026_613_MOESM5_ESM.pdf]

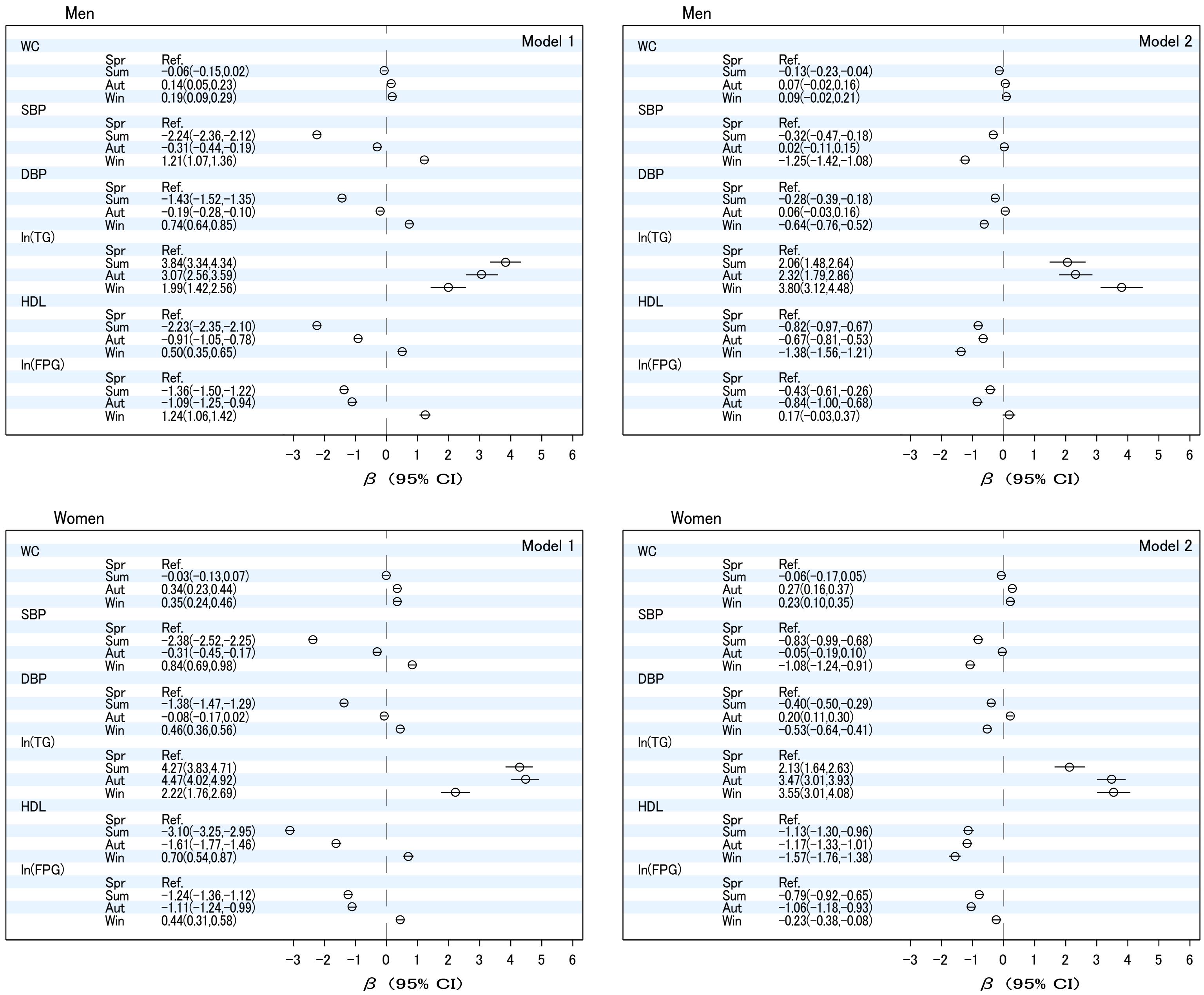

Figure S4. Associations between seasons and metabolic component factors from the GEE analysis, by sex. Forest plots show β coefficients and 95% confidence intervals for seasonal differences in metabolic component factors, using spring as the reference season. Results are shown separately for men and women and for Model 1 and Model 2. Model 1 was adjusted for covariates described in the Methods, and Model 2 was further adjusted for meteorological factors. WC, waist circumference; SBP, systolic blood pressure; DBP, diastolic blood pressure; HDL-C, high-density lipoprotein cholesterol; ln(TG), natural log-transformed triglycerides; ln(FPG), natural log-transformed fasting plasma glucose.
