# Supplementary material for: Seasonal Variation in Metabolic Syndrome and Its Association With Meteorological Factors: A Large Population-Based Study in Fukushima, 2012–2022
Source: J Epidemiol Glob Health. 2026 Jul 9;16(1):126. doi: 10.1007/s44197-026-00613-z (PMC13638822; doi:10.1007/s44197-026-00613-z)
Supplement: Supplementary file 10 [file 44197_2026_613_MOESM8_ESM.pdf]

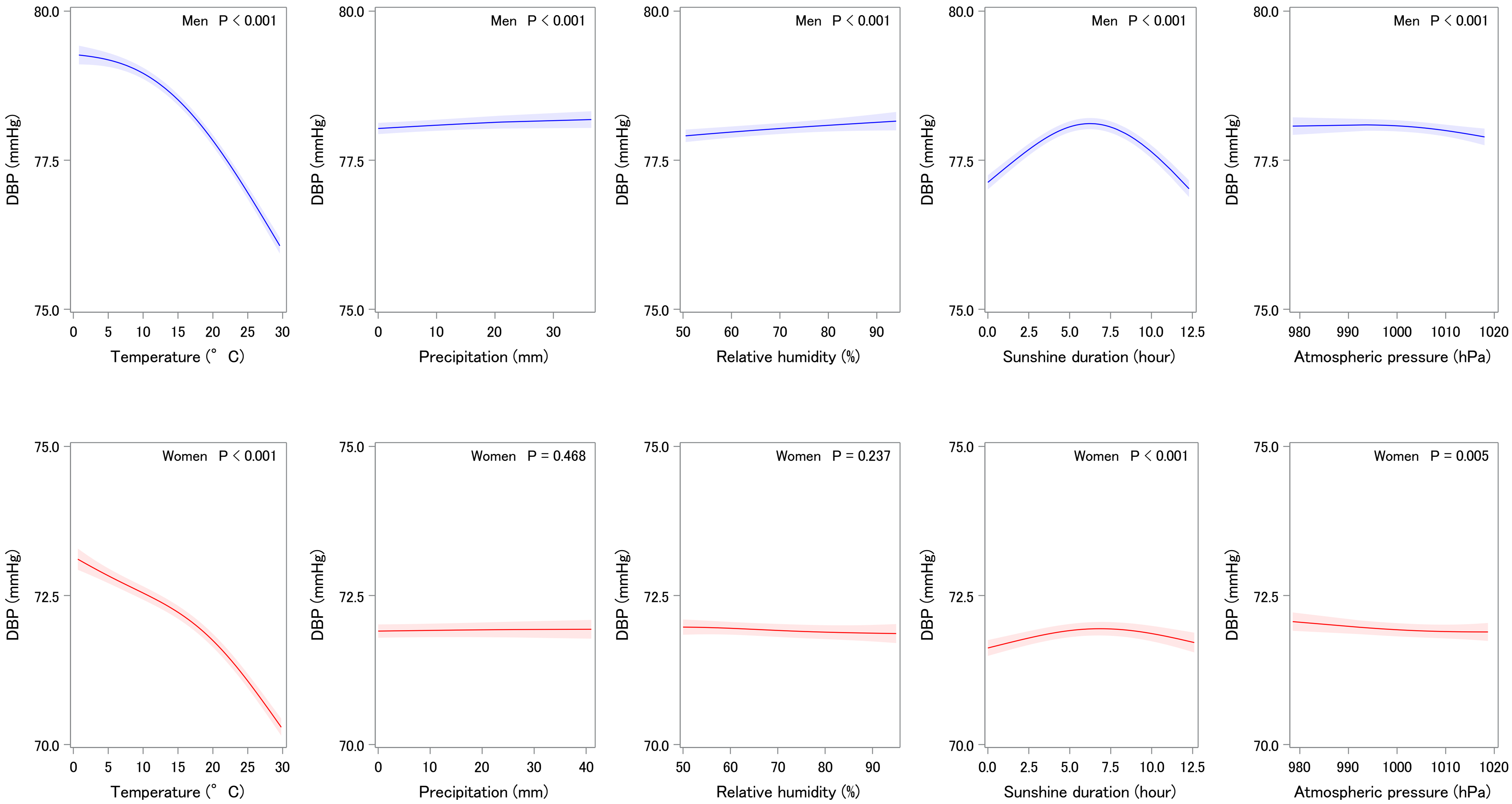

Figure S7. Predicted diastolic blood pressure (DBP) according to meteorological factors from the GAM analysis, by sex. Curves show sex-specific predicted DBP values across the observed range of each meteorological factor. Shaded areas indicate 95% confidence intervals, and P values indicate the statistical significance of each smooth term. Models were adjusted for covariates described in the Methods.
