# Supplementary material for: Seasonal Variation in Metabolic Syndrome and Its Association With Meteorological Factors: A Large Population-Based Study in Fukushima, 2012–2022
Source: J Epidemiol Glob Health. 2026 Jul 9;16(1):126. doi: 10.1007/s44197-026-00613-z (PMC13638822; doi:10.1007/s44197-026-00613-z)
Supplement: Supplementary file 12 [file 44197_2026_613_MOESM12_ESM.docx]

**Table S1.** Spearman correlation coefficients among meteorological factors.

|  | Temperature (^0^C) | Relative humidity (%) | Precipitation (mm) | Atmospheric pressure (hPa) | Sunshine duration (hour) |
| --- | --- | --- | --- | --- | --- |
| Temperature (^0^C) | - |  |  |  |  |
| Relative humidity (%) | 0.316* | - |  |  |  |
| Precipitation (mm) | 0.159* | 0.447* | - |  |  |
| Atmospheric pressure (hPa) | -0.539* | -0.111* | -0.251* | - |  |
| Sunshine duration (hour) | 0.057* | -0.704* | -0.359* | 0.038* | - |

* *P* < 0.001
