# Supplementary material for: Seasonal Variation in Metabolic Syndrome and Its Association With Meteorological Factors: A Large Population-Based Study in Fukushima, 2012–2022
Source: J Epidemiol Glob Health. 2026 Jul 9;16(1):126. doi: 10.1007/s44197-026-00613-z (PMC13638822; doi:10.1007/s44197-026-00613-z)
Supplement: Supplementary file 13 [file 44197_2026_613_MOESM13_ESM.docx]

| **Table S2.** Smooth-component statistics for associations between moving-average meteorological factors and metabolic syndrome from the GAM analysis, by sex. | | | | | | | | | | | | |
| --- | --- | --- | --- | --- | --- | --- | --- | --- | --- | --- | --- | --- |
|  |  | Moving average 0-3 days | | | | |  | Moving average 0-7 days | | | | |
| Sex | Spline Components | EDF | Chi-Square | *P*-value | No of knots | *maxdf* |  | EDF | Chi-Square | *P*-value | No of knots | *maxdf* |
| Men | Temperature (^0^C) | 1.72 | 151.46 | <0.001 | 119 | 3 |  | 1.69 | 125.13 | <0.001 | 119 | 3 |
|  | Precipitation (mm) | 1.99 | 26.05 | <0.001 | 151 | 3 |  | 1.99 | 24.93 | <0.001 | 153 | 3 |
|  | Relative humidity (%) | 1.95 | 244.77 | <0.001 | 120 | 3 |  | 1.95 | 231.08 | <0.001 | 120 | 3 |
|  | Sunshine duration (hour) | 2.00 | 81.82 | <0.001 | 119 | 3 |  | 2.00 | 75.56 | <0.001 | 119 | 3 |
|  | Atmospheric pressure (hPa) | 1.86 | 16.93 | <0.001 | 119 | 3 |  | 1.89 | 21.75 | <0.001 | 119 | 3 |
|  |  |  |  |  |  |  |  |  |  |  |  |  |
| Women | Temperature (^0^C) | 2.88 | 99.02 | <0.001 | 119 | 4 |  | 2.87 | 70.40 | <0.001 | 119 | 4 |
|  | Precipitation (mm) | 1.97 | 21.23 | <0.001 | 151 | 3 |  | 1.97 | 19.41 | <0.001 | 153 | 3 |
|  | Relative humidity (%) | 2.06 | 180.65 | <0.001 | 120 | 4 |  | 2.05 | 174.52 | <0.001 | 120 | 4 |
|  | Sunshine duration (hour) | 2.00 | 57.10 | <0.001 | 119 | 3 |  | 2.00 | 50.94 | <0.001 | 119 | 3 |
|  | Atmospheric pressure (hPa) | 1.88 | 20.42 | <0.001 | 119 | 3 |  | 1.89 | 24.41 | <0.001 | 119 | 3 |

*Models were adjusted for age group (35-39, 40-44, 45-49, 50-54, 55-59, 60-64, 65-69, and 70-74 years), current smoking (no, yes), alcohol drinking (almost no, sometimes, and every day), evacuation (no, yes), resident areas (mountain, central, coastal, and other prefectures), weekly moderate exercises (≥2 times, ≥1 hour/session; no, yes), daily physical activity or walking (≥1 hours/day; no, yes), hypertension, hyperlipidemia, or diabetes therapy (no, yes), history of cardiovascular or kidney disease (no, yes), and seasons (spring, summer, autumn, and winter).
