# Supplementary material for: Seasonal Variation in Metabolic Syndrome and Its Association With Meteorological Factors: A Large Population-Based Study in Fukushima, 2012–2022
Source: J Epidemiol Glob Health. 2026 Jul 9;16(1):126. doi: 10.1007/s44197-026-00613-z (PMC13638822; doi:10.1007/s44197-026-00613-z)
Supplement: Supplementary file 14 [file 44197_2026_613_MOESM14_ESM.docx]

| **Table S3.** Smooth-component statistics for associations between same-day meteorological factors and metabolic syndrome from the season-stratified GAM analysis, by sex. | | | | | | | | | | | | |
| --- | --- | --- | --- | --- | --- | --- | --- | --- | --- | --- | --- | --- |
|  |  | Men | | | | |  | Women | | | | |
| Season | Spline Components | EDF | Chi-Square | *P*-value | No of knots | *maxdf* |  | EDF | Chi-Square | *P*-value | No of knots | *maxdf* |
| Spring | Temperature (^0^C) | 1.28 | 67.45 | <0.001 | 247 | 3 |  | 2.00 | 25.14 | <0.001 | 246 | 3 |
|  | Precipitation (mm) | 1.00 | 1.41 | 0.235 | 80 | 3 |  | 1.00 | 0.19 | 0.665 | 82 | 3 |
|  | Relative humidity (%) | 2.00 | 7.09 | 0.029 | 99 | 3 |  | 1.00 | 0.01 | 0.936 | 99 | 3 |
|  | Sunshine duration (hour) | 1.33 | 0.50 | 0.777 | 163 | 3 |  | 1.83 | 5.20 | 0.074 | 162 | 3 |
|  | Atmospheric pressure (hPa) | 1.00 | 12.95 | <0.001 | 269 | 3 |  | 1.89 | 11.87 | 0.003 | 266 | 3 |
|  |  |  |  |  |  |  |  |  |  |  |  |  |
| Summer | Temperature (^0^C) | 1.75 | 12.03 | 0.002 | 182 | 3 |  | 1.02 | 20.43 | <0.001 | 182 | 3 |
|  | Precipitation (mm) | 1.00 | 0.004 | 0.957 | 113 | 3 |  | 1.00 | 3.06 | 0.0.0 | 113 | 3 |
|  | Relative humidity (%) | 2.00 | 21.23 | <0.001 | 80 | 3 |  | 1.95 | 30.46 | <0.001 | 80 | 3 |
|  | Sunshine duration (hour) | 1.97 | 34.67 | <0.001 | 166 | 3 |  | 1.00 | 6.25 | 0.012 | 166 | 3 |
|  | Atmospheric pressure (hPa) | 1.47 | 0.97 | 0.615 | 225 | 3 |  | 2.00 | 12.11 | 0.002 | 225 | 3 |
|  |  |  |  |  |  |  |  |  |  |  |  |  |
| Autumn | Temperature (^0^C) | 1.02 | 15.40 | <0.001 | 264 | 3 |  | 1.00 | 51.78 | <0.001 | 264 | 3 |
|  | Precipitation (mm) | 1.00 | 5.64 | 0.018 | 100 | 3 |  | 1.00 | 5.33 | 0.021 | 101 | 3 |
|  | Relative humidity (%) | 1.49 | 1.54 | 0.463 | 83 | 3 |  | 1.00 | 36.69 | <0.001 | 83 | 3 |
|  | Sunshine duration (hour) | 1.58 | 2.72 | 0.257 | 146 | 3 |  | 1.00 | 13.14 | <0.001 | 147 | 3 |
|  | Atmospheric pressure (hPa) | 1.09 | 0.90 | 0.638 | 272 | 3 |  | 2.00 | 4.63 | 0.099 | 270 | 3 |
|  |  |  |  |  |  |  |  |  |  |  |  |  |
| Winter | Temperature (^0^C) | 1.62 | 12.44 | 0.002 | 166 | 3 |  | 1.00 | 0.05 | 0.824 | 164 | 3 |
|  | Precipitation (mm) | 1.00 | 8.07 | 0.004 | 70 | 3 |  | 1.00 | 3.59 | 0.058 | 69 | 3 |
|  | Relative humidity (%) | 1.02 | 4.58 | 0.032 | 78 | 3 |  | 2.00 | 25.70 | <0.001 | 78 | 3 |
|  | Sunshine duration (hour) | 1.91 | 12.54 | 0.002 | 132 | 3 |  | 1.71 | 7.51 | 0.023 | 132 | 3 |
|  | Atmospheric pressure (hPa) | 1.61 | 2.35 | 0.309 | 295 | 3 |  | 2.00 | 3.65 | 0.161 | 296 | 3 |

*EDF, effective degree of freedom. Models were adjusted for age group (35-39, 40-44, 45-49, 50-54, 55-59, 60-64, 65-69, and 70-74 years), current smoking (no, yes), alcohol drinking (almost no, sometimes, and every day), evacuation (no, yes), resident areas (mountain, central, coastal, and other prefectures), weekly moderate exercises (≥2 times/week, ≥ 1 hour/session; no, yes), daily physical activity or walking (≥1 hour/day; no, yes), hypertension, hyperlipidemia, or diabetes therapy (no, yes), and history of cardiovascular or kidney disease (no, yes).
